# Supplementary material for: Multi-session smart night charging of EVs for accelerated decarbonization of electric mobility
Source: PLoS One. 2026 Apr 22;21(4):e0345748. doi: 10.1371/journal.pone.0345748 (PMC13102234; doi:10.1371/journal.pone.0345748)
Supplement: Attachment — Submitted filename: Rebuttal_Letter.pdf [file pone.0345748.s001.pdf]

Dyson School of Design Engineering
Imperial College London

03rd October 2025

Revision of manuscript PONE-D-25-37660

Dear Editor-in-Chief,

Please find, enclosed, the revision of manuscript PONE-D-25-37660, "*Multi-Session Smart Night Charging of EVs for Accelerated Decarbonization of Electric Mobility*".

In the revised manuscript, we have incorporated new material following careful consideration of the reviewers' comments. This material is highlighted in BLUE in the revised manuscript. (A version without highlighting is also enclosed.)

Below, we respond point-by-point (shown in BLUE) to the reviewers' comments (quoted in black).

We trust that the revised manuscript will be judged to be a significant improvement over the first version, and acceptable now for publication.

We thank you and the reviewers for your time and expertise in considering our work.

Yours sincerely,

Felix Wieberneit
(on behalf of all co-authors)

Reviewer Comments to the Author

Reviewer #1

“The manuscript proposes a multi-period nighttime smart charging strategy based on model predictive control (MPC). Using UK grid carbon intensity data, it reduces carbon emissions by optimizing multi-day charging periods (up to 7 days). Car owners are required to keep their vehicles plugged in at night and charge only on demand (rather than to full capacity). Simulations show that emissions can be reduced by up to 46% compared to random charging. The proposed method breaks through the limitations of single-day optimization and leverages multi-period flexibility to enhance emission reduction potential. Furthermore, it only requires household smart sockets, which has a low hardware threshold. It also incorporates real regional carbon intensity data and verifies the universality of the proposed method. However, the manuscript has certain shortcomings, such as relying on the accuracy of carbon intensity predictions and insufficient quantification of long-term prediction uncertainties. Furthermore, the potential impact of strategy scalability on grid stability is not discussed.

The content of the manuscript is within the scope of the journal and can be of broad interest to readers. However, in terms of specific content, there is still room for improvement. Therefore, I decided to give the decision of major revision. It is recommended that the author properly absorb the reviewers' comments and make corresponding improvements and enhancements.”

We thank the Reviewer for the time and effort which they have dedicated to reviewing the manuscript, and for the constructive comments which they have provided. Our point-by-point responses now follow.

1.
“For the keywords, 'Carbon Intensity', 'Overnight Charging', 'Multi-Session', and 'Flexibility' should be added to attract a broader readership.”

Action: We have now added the recommended keywords to our manuscript.

2 (first part).
“Page 7, 'Electric Vehicles (EVs) are meeting the expectation of significantly reducing greenhouse gas emissions, as their Global Warming Potential (GWP), expressed in gCO_2e/vkm (grams of carbon dioxide equivalent per vehicle-kilometre), is much lower than that of gasoline and diesel Internal Combustion Engine Vehicles (ICEVs) [1].' We cannot say electric vehicles are absolutely zero-carbon emissions. It depends on how the electricity used to charge the batteries is generated. If it relies on traditional thermal power, it still produces significant carbon emissions. A truly green electric vehicle should rely on excess electricity generated by renewable energy, storing it in the battery through the charging process. Therefore, the author needs to explain clearly the relationship and interaction mechanism between electric vehicles and renewable energy.”

We agree with the Reviewer that most of the emissions due to EVs depend on how the electricity used to charge the batteries is generated. Pleased see, below, a figure that illustrates this fact (taken from [1]) along with our elaboration on this matter.

The figure shows that production, maintenance and other minor aspects of the operation of an EV are approximately constant. In contrast, the electricity production cycle strongly depends on the specific country. This has a very low impact in countries with decarbonized energy, e.g. in France (FR, because of nuclear power) and Sweden (SE, because of hydro). Meanwhile, it has a high impact in countries which heavily leverage on higher-emissions fuels (e.g., coal). For instance, the total GWP of EVs in Estonia (EE) is higher than the average for ICEV-G or ICEV-D, due to the average mix from the grid. However, according to this report, the average GWP in Europe is already significantly lower than that of ICEV-G and ICEV-D, and it is supposed to decrease further in the future as the penetration level of energy generated from renewable resources is expected to increase.

Action: In the introduction of the revised manuscript, we have reported and elaborated this point (page 2, lines 10-17), thereby improving the explanation regarding the relationship and interaction mechanism between EV charging and renewable energy.

[1] Report, Study by Fraunhofer ISE & Fraunhofer ISI on behalf of Transport & Environment "Study on the "Potential of a full EV-power-system-integration in Europe & how to realise it"", 2023.

Figure ES5: Comparison of Lower Medium Car lifecycle GWP impacts for conventional gasoline/diesel ICEVs and BEVs for different EU countries, Baseline scenario. Breakdown shown for new 2020 vehicles, and the total only for new 2030 vehicles.

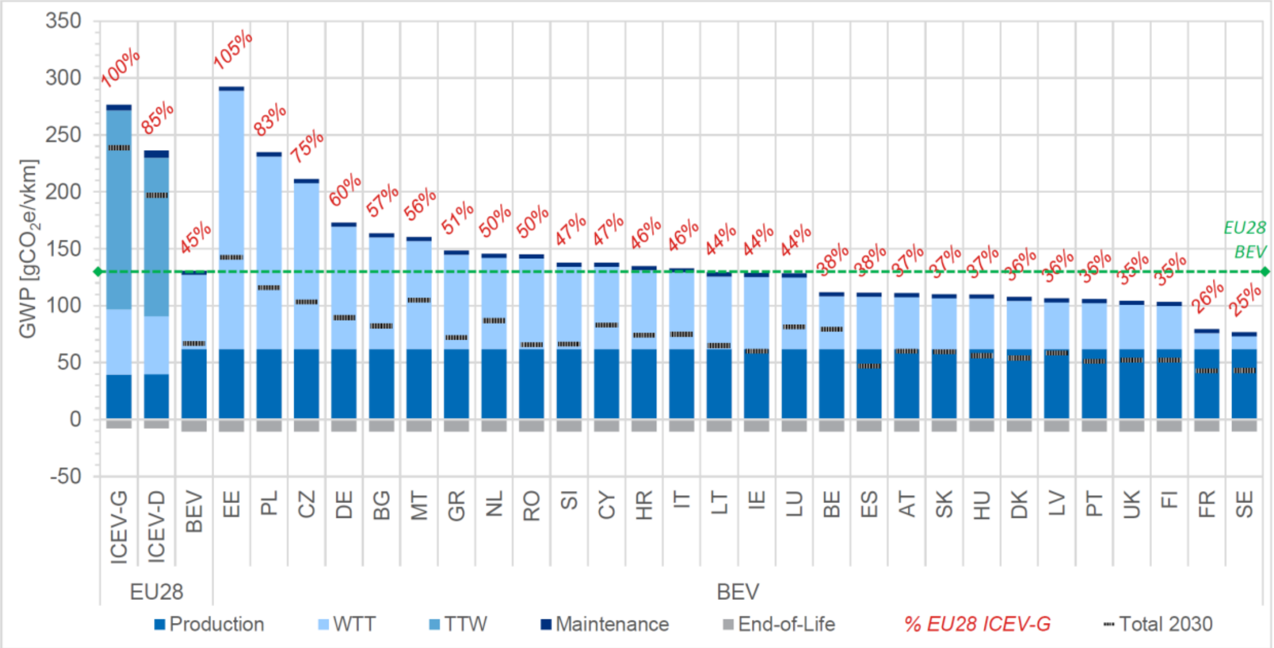

Notes: Results shown for the lower medium car in the baseline scenario. Production = production of raw materials, manufacturing of components and vehicle assembly; WTT = fuel/electricity production cycle; TTW = impacts due to emissions from the vehicle during operational use; Maintenance = impacts from replacement parts and consumables; End-of-Life = impacts/credits from collection, recycling, energy recovery and disposal of vehicles and batteries. Additional information on key input assumptions and derived intermediate data include the following: a lifetime activity of 225,000 km over 15 years. 2020 BEV battery of 58 kWh, with 300km WLTP range (and with 64 kWh and 460 km WLTP electric range for 2030); an average lifetime EU28 fuel/electricity mix (age-dependant mileage weighted). No battery replacement is needed for BEVs.

2 (second part).

"For example, most renewable energy sources are intermittent, opening spatial and temporal gaps between the availability of the energy and its consumption by end users. In order to address these issues, it is necessary to develop suitable energy storage systems for the power grid (10.1016/j.electacta.2019.03.056). Therefore, electric vehicles using lithium batteries have become an important support for the efficient recycling and utilization of renewable energy."

We agree with the Reviewer on this point as well. In fact, our proposed methodology aims at charging the EVs mainly when carbon emissions are low, i.e. at times when more energy is generated from renewable sources. In this way, EVs would, indeed, be used for efficient recycling and utilization of renewable energy, as stated by the Reviewer.

Action: In the revised manuscript (Introduction, page 2, lines 20-24), we have added a comment to clarify that EVs may be used as mobile storage systems for the support of renewable energy utilization.

3.

“The presentation of innovative points requires enhanced comparison. The manuscript emphasizes "the first multi-period optimization for a single EV household scenario," but fails to adequately compare existing research. Therefore, it is recommended to include a literature comparison table in the Introduction, quantifying the marginal contribution of this study compared to single-period optimization (e.g., 24-hour strategies).”

In the revised manuscript, we have substantially extended our review of the state-of-the-art (Introduction, page 2, lines 37–59). We now clarify that while there is extensive literature on smart charging algorithms, most optimize functions of interest other than carbon emissions, such as cost of charging, load balancing, peak load, grid impact, or user convenience.

Studies which explicitly target carbon minimization remain comparatively limited, but we now cite and discuss several relevant contributions in the revised manuscript. For example, Dixon et al. [15] demonstrate that fleet-scale charge scheduling in Scotland can reduce emissions while mitigating wind curtailment; Huber et al. [16] and Mehlig et al. [17] highlight possible emission reductions based on average and marginal emission factors; Li et al. [18] propose a combined carbon emission and time-of-use price objective. These works highlight the growing research interest in controlled charging for carbon emission reduction, yet they typically assume perfect foresight, focus on fleet/aggregator scales, or they optimize only over a single charging session or day.

In contrast, our study addresses the under-explored case of domestic overnight charging with minimal hardware requirements (i.e. a simple smart plug), and it introduces a multi-session MPC framework that explicitly accounts for forecast uncertainty and user behaviour over several days. To make the comparison more transparent, we have also revised Table 2 (page 10) to include a column that quantifies the further contribution (in terms of emission reduction) of our multi-session strategy over single-period (24-hour) approaches.

Action: We have revised the Introduction section of our manuscript with an extended literature review (page 2, lines 37-59), now including additional references [16, 19]. Additionally, in Table 2 (page 10), we have added a column that numerically quantifies the marginal contribution of our multi-session optimization results compared to single-session optimization.

4.

“The theoretical basis for multi-period optimization is weak, and the reason for choosing a 7-day upper limit for the forecast window is not explained. Additional theoretical support is needed,

including reference to time series forecasting theory and a sensitivity analysis demonstrating the saturation point between the window length and emission reduction benefits.”

As per the reviewer’s recommendation, we have provided additional results and discussion (Section 4.1), including a new figure (Fig. 4), to illustrate the saturation point between the forecast window length and emission reduction benefits. These results – which have been obtained via a Monte Carlo sensitivity analysis – demonstrate that increasing the future horizon length beyond 3-4 days does not provide significant improvement in terms of emission reductions. This can be attributed to the uncertainty in the forecasting of future carbon emissions. Thus, the arbitrary choice of a 7-day horizon is sufficient in the sense that it reveals the diminishing benefits of optimization horizons greater than 3-4 nights with respect to emission reduction.

Action: In the revised manuscript, we have added a new figure (see Fig. 4 of the revised manuscript) to evaluate how the emission reduction benefit varies with the choice of future horizon length. We have extended Section 4.1 to elaborate on the data presented in the new figure and to address the reviewer’s comment.

5.

“The full lifecycle analysis of the vehicle energy system needs improvement. The manuscript focuses solely on carbon emissions from the charging phase and fails to consider the impact of the lifespan degradation of the vehicle's energy storage device on the full lifecycle carbon footprint. It is recommended that the "Lifecycle Assessment" section include an analysis of the contribution of energy storage device (battery/fuel cell) durability to carbon emissions, while also introducing a state-of-health (SOH) degradation model to quantify the incremental carbon emissions implicit in long-term use. Authors can refer to 10.1109/TPEL.2024.3502499, which proposes a lifespan prediction framework under dynamic conditions that can be directly transferred to EV battery health management, helping authors construct a coupled SOH-carbon emissions model and enhancing the rigor of the full lifecycle analysis.”

In our work, we do not provide a full lifecycle carbon analysis of the energy storage device (such as the one in the figure above, in response to comment 2). We focus only on the carbon emissions from the charging phase (which are the most relevant for our analysis, being related to generation from renewable sources). Since we compare two different charging strategies strategies (i.e. plain charging and smart unidirectional charging), we assume that the difference in carbon emissions associated with the two strategies is only due to the charging choice.

The reviewer raises an important point, nonetheless: carbon emissions have *direct* and *indirect* sources. In our work, we only analyze the *direct* ones. However, as we will now explain in detail, our approach is also convenient in terms of further reducing *indirect* emissions.

Consider the following outcome of the report [1, page 70], where controlled unidirectional charging had been implemented to minimize the cost of charging: “*the lifetime of the battery can be substantially extended by changing the target SOC upon departure from 100% to 80% instead. This increases the lifetime by an average of 40%. Controlled unidirectional charging increases the battery lifetime by a further 5-10% on average, compared to uncontrolled charging*”.

In our work, we already set the target SOC upon departure to 80% for both scenarios (i.e. smart controlled and uncontrolled charging), specifically with the purpose of extending the lifetime of the battery.

However, as found in [1], smart control has the further advantage of increasing the battery lifetime by a further 5-10% on average (this result has been obtained by embedding both linear and non-linear ageing models, with better results with non-linear ageing models, as shown on page 90 of the report). Quoting from [1]: "*the main source for extended lifetime is the reduced calendrical ageing resulting from more beneficial charging states*". Indeed, if uncontrolled, then charging is from a minimum level to a maximum level, while controlled charging operates more frequently around intermediate levels of the SOC.

Action: In section 2.1.2 (page 6, lines 158-160) of the revised manuscript, we cite reference [2] and discuss the findings there, to support our choice never to charge vehicles to more than 80% of the SOC. We also now highlight that further benefits may be obtained by applying our proposed strategy, specifically that smart charging strategies extend the lifetime of batteries, as explained in [2]. In addition, we clarify that more sophisticated models - such as the linear and nonlinear models of [2] - could be adopted, so as to take into account the degradation of the battery (page 6, lines 169-177).

[1] Report, Study by Fraunhofer ISE & Fraunhofer ISI on behalf of Transport & Environment "Study on the "Potential of a full EV-power-system-integration in Europe & how to realise it"", 2023.

6.

"The carbon intensity forecasting model is insufficiently disclosed. Formula (12) briefly mentions it as a "1-step-ahead forecast," but does not specify the specific forecasting algorithm (ARIMA/LSTM/Transformer), the input variables (whether covariates such as weather and electricity prices are considered), or the quantitative results of the forecast error (it is recommended to add MAPE/RMSE indicators)."

In our study, we do not develop a carbon intensity forecasting model, but, instead, construct synthetic long-range forecasts from the publicly available UK National Energy System Operator (NESO) one-step-ahead forecasts. The NESO data provide actual carbon intensity values and corresponding one-step-ahead forecasts only. No multi-step forecasts are stored. To model the longer-range forecasts required by our optimization, we therefore synthesize multi-step-ahead forecasts by applying error growth rates estimated from the published *CarbonCast* benchmark study [22] (i.e. [20] in the original manuscript). This procedure allows us to account for the empirically observed growth of forecast uncertainty with horizon length, without assuming a specific forecasting model such as ARIMA, LSTM, or Transformer.

Action: We have revised Section 3.1 to emphasize that our objective in that section is not to provide a forecasting model, but, rather, to synthesize realistic long-range forecasts based on the available one-step ahead forecast sequence (NESO) and the published long-range error statistics (page 7, lines 197-199). Furthermore, we now include quantitative error statistics for both NESO-published one-step-ahead forecast errors (Fig. 2) and for the synthesized long-range forecast (Fig. 3).

7.

“The user behavior modeling is overly idealistic. The assumption that users "only request the required amount of electricity each day" fails to account for behavioral uncertainty, requiring the introduction of stochastic programming or robust optimization. The authors could further analyze the impact of temporary increases in user travel on emissions reductions, and appropriately cite behavioral economics research.”

We thank the Reviewer for the opportunity to clarify the misunderstanding here. We agree that the quoted phrase from the Abstract miscommunicated the behavioral modelling. In fact, our modelling does account for uncertainty in user behavior (specifically in the realized plug-in and plug-out times as well as in daily energy demand), as explained below.

We have extracted mean daily energy demand and day-to-day variability from real-world data [25] (which was [21] in the original manuscript). In our simulations, realized daily energy demand values are sampled from a gaussian distribution which is calibrated based on this real-world data, reflecting typical uncertainty in daily energy demand of EV drivers.

However, we then assume that the user communicates to the system a precautionary (upper bound) estimate of their typical daily energy requirement (corresponding to the 98th percentile value of actual energy demand). This ensures that expected energy requirements are typically much greater than realized values. In this way, we model over-cautious users. We note that greater benefits could be obtained if users were, indeed, able to predict their energy consumption in a more accurate (and less conservative) way. In summary, our work prioritizes the uncertain energy needs of the drivers and we are conservative in estimating their values.

Action: In the abstract, we have explicitly stated that users communicate a precautionary estimate of their travel demand. Then, in Section 3.3, we clarify how this estimate is computed, and we note there that better results could be obtained if more accurate estimates were provided to the charging system (page 9, lines 282-286).

8.

“The potential impact of reversible losses on the scheduling strategy is overlooked. The manuscript assumes constant charging efficiency, but real-world batteries/fuel cells exhibit reversible voltage loss recovery, which can affect the accuracy of multi-period optimized power allocation. It is recommended to add a dynamic model for reversible losses to the MPC constraints and analyze the sensitivity of voltage recovery characteristics to the nighttime segmented charging strategy. The authors are referred to the Journal of Power Sources 625 (2025): 235634. The voltage recovery quantitative model established there can help the authors improve the objective function (Equation 7) and enhance the physical feasibility of the strategy by embedding a correction term in the carbon emission optimization.”

We agree with the Reviewer that details such as a dynamic model accounting for reversible voltage losses in battery charging could be added of our work. However, such a level of detail is rarely used in analyses similar to the ones we carry out in our work, in which slow charging is considered, involving slots of at least 30 minutes.

Action: In section 2.1.2 (page 6, lines 169-177) of the revised manuscript, we emphasize that the main focus of our work is the comparison between uncontrolled and smart charging (with the objective of minimizing carbon emissions). We also now discuss a more sophisticated dynamic model of the charging of the battery as a future extension, so as (i) to enhance the accuracy of our analysis and for closer correspondence with reality, and (ii) to verify the findings of other works which claim a further improvement in battery lifetime if smart charging techniques are adopted instead of uncontrolled charging (see also Action 5 above).

9.

"Verification of MPC's real-time performance is lacking. The computational complexity of the optimization problem is not specified, nor is the solution time for a single EV sufficient for real-time control. Computational platform configuration and time consumption data are needed.) Furthermore, a brief comparison of the efficiency differences between commercial solvers (e.g., Gurobi vs. CPLEX) is recommended."

The run time for the proposed optimization - even on a standard laptop - is extremely small (<1 s for $N \leq 7$), and there are no computational performance issues for real-time implementation of the proposed scheme for a large population of users. We used the open-source Clarabel solver (from CVXPY) for solving the optimization problem. Since the computational time is not an issue, the difference between the performance of Clarabel, Gurobi or CPLEX would be negligible for our purposes.

Action: In the revised manuscript, in section 2.1.2 (page 6, lines 178-183), we now provide a remark on the computational performance of the proposed optimization, including the computation time using CVXPY with the Clarabel solver on a standard laptop (Apple M2).

10.

"The generalizability of the 46% emission reduction conclusion is questionable. First, the results only present single-week data (Figure 1), failing to verify robustness across seasons and years. Second, additional boxplots are needed to illustrate the distribution of emission reduction rates across the entire year (by region and season). Finally, the risk of policy failure in extreme cases (such as high carbon intensity during cold waves) is largely undiscussed. These issues require further clarification and refinement."

We thank the Reviewer for this opportunity to provide complementary results and clarification. In the original manuscript, our results were obtained over a 4-month horizon. However, in the revised version, we have extended this horizon to a full year of simulation, to capture better the seasonality effects, as suggested by the Reviewer.

It can now be observed that improvements are consistent throughout the year. However, the improvement over the whole year - averaged over ten random independent realizations of the scenario - has decreased from 46% to about 37%.

Action: We have extended the temporal scope of our simulation from four months to a whole year and included a seasonality analysis in Section 4.1.1 (page 10, lines 315-324). We have also included a new figure (Figure 5), which demonstrates the consistent performance of the proposed algorithm across all months of the year.

Reviewer #2

“This paper proposes an optimization strategy for multiple nighttime charging of electric vehicles based on multi-period predictive control to reduce carbon emissions. The paper's ideas are clear, the experimental data is comprehensive, and the results demonstrate significant reductions in carbon intensity, demonstrating strong engineering application value. However, the following issues require improvement:”

We thank the Reviewer for their time and effort in reviewing our manuscript, for their acknowledgement of its merits, and for their constructive comments. Next, we provide a point-by-point response to the Reviewer’s comments, and we specify the actions we took in each case to improve the manuscript.

1.
“The introduction provides a comprehensive review of related work, but insufficient coverage of research on uncertainty in carbon intensity predictions. A literature comparison should be included.”

In the revised manuscript, we have expanded the literature review in order to address the research on uncertainty in carbon intensity forecasting. We now discuss recent studies showing that machine learning approaches can achieve very accurate short-term forecasts (up to six hours ahead) [20], while longer horizon forecasts (up to four days ahead) are feasible but with increasing uncertainty with prediction horizon (MAPE of 5–14% depending on region) [21]. We further reference recent contributions that emphasize probabilistic and uncertainty-aware approaches, including probabilistic interval forecasts [22] and a review of statistical metrics for quantifying forecast uncertainty [23].

Together, these additions highlight the fact that forecast uncertainty depends on horizon length, methodological approach, and regional factors, and that state-of-the-art forecasting methods today can provide both accurate point predictions and calibrated uncertainty intervals. We believe this extended discussion strengthens the Introduction and directly addresses the Reviewer’s concern.

Action: We have revised the Introduction section of our manuscript by including a review of recent research on the uncertainty in carbon intensity predictions (page 2, lines 47-60). We have cited three additional references [20,22,23] in addition to the previously referenced long-horizon forecast method [21].

2.
“The "46% emission reduction" result mentioned in the abstract should be clarified under what circumstances.”

The 46% emission reduction was obtained in a simulation over 4 months. We used carbon emissions data for the UK. A different country - with different levels of renewable energy penetration – would obviously yield different improvements. Note that if a country has constant carbon emissions per unit of energy (e.g. because only a single source of power generation is used), then our algorithm would not lead to any improvements, would be constant over time and scheduling charging would not affect the carbon intensity of the electricity consumed.

Action: nIn the revised manuscript, we have made a number of revisions related to this point:
Firstly, we have extended the length of the simulation from 4 months to a full year;
Secondly, we are now averaging the results over 10 Monte Carlo simulations, to smooth out the stochastic realizations (e.g. variability user behavior, or carbon emissions forecasts).
Finally, we now consider seasonality aspects.
Due to this more thorough analysis, the reported overall reduction in carbon emissions over the one-year period has decreased to 37% (it was 46% over the 4-month period), but we show that our proposed method is consistently effective in reducing charging emissions throughout the year (see Fig. 5 and Section 4.1.1).

3.
"The linear growth assumption (Equations 10-12) is used in the modeling of carbon intensity prediction errors. Its rationale and potential limitations should be further explained."

The assumption of linear growth in forecast error with horizon length is a modelling choice motivated by existing, published literature on long range carbon intensity forecasting [22] (i.e. [20] in the original manuscript). The cited study reports mean absolute percentage error (MAPE) values up to four days ahead across multiple regions. These MAPE values exhibit an approximately linear—though slightly concave—relationship with the horizon length. We adopt the simplified linear form because (i) it captures the dominant error growth trend observed in published data, (ii) it provides a conservative estimate by slightly overestimating long-range error magnitudes, and (iii) it allows a transparent, analytically tractable way of embedding forecast uncertainty in the evaluation of our optimization framework.

We acknowledge, however, that the linear assumption has limitations. In particular, (a) error growth may saturate or follow nonlinear patterns depending on system conditions, renewable penetration, and forecasting methodology; and (b) the use of region-averaged error growth rates (rather than UK-specific values) introduces uncertainty in the calibration of the error growth rate.

Action: We have added a discussion of these points in Section 3.1.2 (page 8, lines 231-235), noting that future work could refine the error model using UK-specific multi-step forecast data and/or by adopting a nonparametric modelling technique. We have also added a new figure (Figure 3) to illustrate the linear error growth in our synthesized carbon intensity forecasts.

4.
"It is recommended to add a table summarizing the key symbols and their physical meanings to facilitate reader understanding."

Action: In the revised manuscript, we have added a table summarizing the items of notation and their physical meaning (Table 1 in Section 2).
