# Supplementary material for: Multi-session smart night charging of EVs for accelerated decarbonization of electric mobility
Source: PLoS One. 2026 Apr 22;21(4):e0345748. doi: 10.1371/journal.pone.0345748 (PMC13102234; doi:10.1371/journal.pone.0345748)
Supplement: Attachment — Submitted filename: Round_2_Rebuttal_v2-1.docx [file pone.0345748.s002.docx]

**Responses to Reviewer Comments**

**Reviewer #1:**

**This paper proposes an innovative multi-stage MPC charging strategy to reduce the carbon intensity of overnight charging of electric vehicles. The experimental design is rigorous, and the results have practical value (e.g., a 37% carbon emission reduction). However, there is room for improvement in terms of hardware efficiency and future technology integration. Here are a few suggestions:**

**1.1. The manuscript assumes constant charging efficiency, but energy losses (such as conversion losses) occur during the actual charging process, which are not modeled. In particular, the manuscript relies on "household smart plugs" as the hardware foundation, which may limit the practical applicability of the strategy. For example, compatibility issues may lead to user inconvenience or reduced efficiency.**

**It is recommended to briefly discuss the hardware constraints of wired charging (such as plug compatibility) in the "Introduction" or "Discussion" section. Recent research should be cited to emphasize how charging interoperability improves overall efficiency. For example, reference [10.1109/TPEL.2024.3429535] shows that optimizing wireless charging interoperability (such as defining impedance boundaries) can reduce energy losses, which is highly synergistic with the carbon reduction goals of this study. The quantitative methods proposed in the literature to assess charging efficiency can help demonstrate the indirect contribution of "hardware optimization" to carbon reduction.**

**Author Response**

We thank the reviewer for highlighting charging-efficiency losses and practical implementation constraints. We agree that the original model did not take into account charging efficiency and did not explicitly represent conversion and auxiliary losses (EVSE, cable, and on-board charger). To address this, we have revised the formulation to include a charging-efficiency parameter $\eta\in\left( 0,1 \right]$ in the SOC dynamics, capturing that only a fraction of the grid-supplied energy $P\left[ s,k \right]\Delta_{t}$ ends up stored in the battery.

Our contribution remains algorithmic: it is a multi-session MPC scheduling layer that minimizes emissions using carbon-intensity forecasts, and it is largely independent of the charging modality, provided charging power can be actuated (continuously via a controllable EVSE, or approximately via time-averaged on/off switching). Under the (standard) approximation that $\eta$ is approximately constant for domestic AC charging over the considered SOC range (20–80%) and common across the compared strategies, including $\eta$ primarily rescales SOC evolution and absolute energy flows but does not materially change the relative comparison between uncontrolled charging, single-session MPC, and multi-session MPC.

Regarding the suggested reference: We agree that charging hardware interoperability affects losses during the charging process, however, given that the present work deliberately focusses on the temporal control dimension, we feel that a detailed discussion of impedance-space interoperability for wireless systems would be out of scope. However, we appreciate the broader point that interoperability and hardware efficiency are complementary levers to reduce lifecycle emissions.

**Author Action**

- We have updated the SOC charging dynamics (Eq. 3) to include a charging-efficiency parameter $\eta$, and added $\eta$ to the notation table.
- We have included a short remark (Model Accuracy / Practical Implementation) stating that we neglect detailed power-electronics/thermal effects and treat $\eta$ as approximately constant; noting that more detailed efficiency models can be incorporated without changing the proposed control framework.
- We have added a brief clarification that the control approach is hardware-agnostic, requiring only an interface to actuate charging power (or its time-averaged equivalent via on/off control), and that the reported comparisons assume the same $\eta$ across strategies.

**2. Expand future research directions to include the potential of wireless charging. The current manuscript focuses on wired charging, but wireless charging is a key trend in the electrification transition. Ignoring this point may weaken the manuscript's long-term impact.

It is suggested to add a paragraph in the "Conclusion" or "Future Work" section to discuss the possibility of multi-technology integration. For example: Future research could explore the integration of intelligent scheduling strategies with wireless charging systems, where interoperability standards (Energies 16.4 (2023): 1653) can further improve user convenience and system efficiency. This literature summarizes the role of interoperability in promoting energy recovery, directly echoing the theme of this manuscript, "Utilizing Excess Electricity from Renewable Energy Sources."**

**Author Response**

We agree that wireless charging technologies are a relevant long-term development in EV infrastructure and that interoperability standards for such systems can enhance user convenience and system efficiency. Our MPC framework is agnostic to the physical interface of the charging system; it optimizes power trajectories given availability windows and forecasted carbon intensity. In principle, the same logic could be applied to wireless systems.

At the same time, the present paper is deliberately scoped to a very simple and widely deployable use case—domestic overnight charging via a conventional smart plug. We therefore prefer to keep wireless charging clearly identified as future work, rather than expanding the technical content in that direction.

**Author Action**

- In the Conclusions we added a short paragraph stating that:
  - Although the case study is scoped for domestic charging with smart plugs, the proposed multi-session MPC framework is hardware agnostic and could be interfaced with other technologies such as wireless charging.

**Reviewer #2:**

**My review comments have been fully addressed and resolved by the authors. They have carefully revised the manuscript according to the feedback, and the revised version satisfactorily answers all the concerns raised during the review process. The quality and clarity of the paper have been significantly improved as a result of these revisions. No further major issues remain to be discussed. Therefore, I believe the manuscript now meets the publication standards of the journal and can be accepted in its current form.**

**Author Response**

We thank the reviewer for their feedback and are pleased that the revision addressed the earlier concerns.

**Reviewer #3:**

**In this paper, authors presented a multi period predictive control based optimization approach for multiple nighttime electric vehicle charging stations with the goal of lowering carbon emissions. The experimental data is thorough, the paper's concept is straightforward, and the findings show notable decreases in carbon intensity. Considering all these points, the article is now recommended for acceptance.**

**Author Response**

We thank the reviewer for their feedback and are pleased that the revision addressed the earlier concerns.

**Reviewer #4:**

**The majority of electric vehicles (EVs) are charged domestically overnight, when the precise timing of power allocation is not important to the user, thus providing a source of flexibility that can be leveraged by charging control algorithms; This is addressed by the authors.**

**Dear Author,**

**I do appreciate your efforts for a well revision.**

**Best wishes,**

**Dr. V B Murali Krishna**

**Author Response**

We thank the reviewer for their feedback and are pleased that the revision addressed the earlier concerns.

**Reviewer #5:**

**The revised version of the paper is acceptable for publication . All the comments have been addressed by the authors .**

**Author Response**

We thank the reviewer for their feedback and are pleased that the revision addressed the earlier concerns.

**Reviewer #6:**

**This manuscript explores model predictive control for multi-session EV charging to minimize carbon emissions, leveraging UK grid data and user flexibility, which addresses a timely issue in sustainable mobility. The simulations demonstrate emission reductions, but the assumptions on user behavior and forecast accuracy are overly optimistic without sufficient sensitivity analysis. While valuable, the paper requires major revisions to enhance realism, expand comparisons, and refine the optimization framework prior to acceptance.**

**1. The introduction claims EVs reduce GWP significantly, but does not discuss variability across grids with high fossil fuel reliance. Elaborate on this with data from diverse regions to contextualize the UK focus.**

**Author Response**

We agree that grid carbon intensity varies widely across regions and that this affects both the absolute and relative lifecycle benefits of EVs. In the revised introduction we already expanded the discussion to emphasise that:

- Use-phase GWP is highly contingent on the generation mix, and the same EV can have very different lifecycle GWP depending on local grid carbon intensity;
- Some countries/regions with predominantly fossil-based generation (e.g., Estonia) show much higher use-phase GWP than predominantly fossil fuel grids (e.g., France or Sweden).

To respond more explicitly to the reviewer’s concern, we now:

- Clarify that the UK is used as a case study with rich, publicly available half-hourly carbon intensity data;
- Note that achievable emission reductions are contingent on the specific carbon intensity sequence of the electricity grid.

**Author action**

- In the Introduction, we refined the paragraphs discussing lifecycle GWP and grid carbon intensity to:
  - Explicitly highlight the variability between low-carbon and high-fossil grids;
  - Clarify that the UK is used because of the availability of high-resolution carbon intensity data;
  - State that the same MPC framework can be applied to grids with different carbon profiles, so long as the carbon intensity is not constant, with absolute savings depending on the average carbon intensity and its variability over time.

**2. The MPC formulation for multiple sessions is clear, but constraints on battery degradation are omitted. Incorporate state-of-health models to make the approach more practical.**

**Author response**

We agree that explicit state-of-health modelling is important for long-term operational planning and that there is a natural coupling between carbon-optimal charging and degradation-optimal charging. In this work, we adopt a simple surrogate for battery state of health:

- SOC is constrained to [20%, 80%], in line with recent reports indicating that limiting SOC to this range can substantially extend battery lifetime;
- We focus on the *relative* difference between uncontrolled and smart charging under this SOC policy.

We have already acknowledged in a Remark (Model Accuracy) and in the Conclusions that we neglect SOC-dependent efficiency and battery ageing, and cited recent work showing that smart charging can extend battery lifetime. In response to the reviewer’s request, we now emphasise more clearly that:

- Our SOC constraints are chosen to be consistent with reduced degradation;
- Integrating an explicit SoH model into the MPC (e.g., via additional state dynamics or cost terms) is an important but non-trivial extension and is left to future work to avoid over-complicating the current framework.

**Author action**

- In Section 2.1.2, in the Remark (Model Accuracy), we added explicit clarification that:
  - SOC bounds [20%, 80%] are chosen to reflect commonly recommended operating ranges for reduced degradation.
  - Ageing and detailed SoH dynamics are not modelled in the current optimisation.
- In the Conclusions, we strengthened the paragraph discussing battery lifetime to:
  - Highlight that maintaining SOC in reduced ranges may lower indirect emissions by delaying battery replacement.
  - Explicitly identify the integration of coupled SoH–carbon models within the MPC as a key avenue for future work.

**3. The literature on smart charging is reviewed, but recent multi-period optimizations are underrepresented. To improve the methodological discussion and incorporate advanced flexibility strategies, consider these papers: Optimal scheduling of electric vehicle charging operations considering real-time traffic condition and travel distance, Expert Systems with Applications; Composite Neural Learning-Based Adaptive Actuator Failure Compensation Control for Full-State Constrained Autonomous Surface Vehicle, Neural Computing and Applications; Saturated-threshold event-triggered adaptive global prescribed performance control for nonlinear Markov jumping systems and application to a chemical reactor model, Expert Systems with Applications. This will help authors refine forecast windows and emission minimization.**

**Author response**

We thank the reviewer for suggesting additional references.

After reviewing the proposed papers, we decided not to include them as citations because they do not directly inform the modelling assumptions or optimisation structure used in this manuscript.

The EV scheduling paper (Expert Systems with Applications: “Optimal scheduling of electric vehicle charging operations considering real-time traffic condition and travel distance”) studies urban/public-charging operations with routing/traffic and queueing/assignment decisions, with the objective of minimizing total charging time. Charging power is treated as a fixed parameter rather than a controllable trajectory, and the formulation does not address carbon-intensity–driven objectives or multi-session domestic overnight charging. We therefore believe citing it would not materially clarify our MPC formulation, forecast-window design, or emissions-minimisation focus.

The other two suggested papers address adaptive/event-triggered control in unrelated domains (autonomous surface vehicles and nonlinear Markov jumping systems applied to a chemical reactor). While methodologically interesting, their control architectures and application contexts are not used here and would be considered irrelevant to the smart-charging literature reviewed in this work.

**Author action**

Instead of adding these citations, we strengthened the Introduction/Literature discussion to more explicitly position our contribution relative to multi-period EV charging optimisation: we clarify that our novelty is a multi-session, carbon-aware MPC scheduling layer for domestic overnight charging, and we avoid expanding the scope to routing/queueing-based public-charging formulations.

**4. Simulations use 2022 UK data, but lack scenarios with renewable variability like wind intermittency. Add stochastic elements to test robustness.**

**Author Response**

We appreciate this important point on robustness to renewable variability. In our study, the carbon-intensity input is the half-hourly historical UK grid carbon intensity for 2022, which already embeds real renewable variability (including wind intermittency) and its interaction with demand and conventional generation. In addition, for the annual results we perform multiple Monte Carlo runs with randomised user sequences (plug-in times and daily energy consumption) and report mean and standard deviation across these runs. The controller is therefore evaluated under both realistic grid variability and behavioural variability.

We agree that this was not sufficiently emphasised in the original manuscript, and we are grateful for the suggestion to make the robustness aspect more explicit.

**Author action**

We have revised Section 3 to clarify that:

(i) the simulations use full-year, half-hourly historical UK carbon-intensity data, which inherently captures renewable intermittency, and especially wind intermittency; and

(ii) all annual results are based on multiple Monte Carlo runs with randomised user behaviour, with mean and standard deviation reported as a robustness indicator.

**5. The 37% reduction is impressive, but not benchmarked against single-session MPC. Include direct comparisons to highlight multi-session advantages.**

**Author response**

We agree that explicitly highlighting the benefit of multi-session MPC over single-session MPC is important. In the current results section and Table 2, we already:

- Report the carbon intensity C_{EV} for uncontrolled charging (N=0), single-session MPC (N=1), and multi-session MPC for N=2,…,7.
- Quantify the improvement of each N relative to N=1 (single-session MPC).

However, we recognise that this comparison may be somewhat buried in the text and table. To address this, we now make the relative improvement over single-session MPC more prominent in the narrative.

**Author action**

- In Section 3.1 (Annual Emission Reductions with N-day MPC Charging), we added one explicit sentence summarising that:
  - In our 2022 UK case study, N=4 multi-session MPC reduces the average carbon intensity C_{EV} by approximately 23% relative to an otherwise identical N=1 single-session MPC baseline.
- In the Conclusions, we added a short phrase explicitly stating that multi-session MPC outperforms both uncontrolled charging and single-session MPC, and we again quote the representative relative improvements.

**6. User flexibility assumptions (e.g., precautionary energy estimates) need empirical support. Reference surveys or add a section on incentive mechanisms.**

**Author Response**

Our modelling of user behaviour is data-informed but deliberately simplified and conservative. Plug-in / plug-out times and daily energy consumption are calibrated based on the UK Department for Transport’s Electric Chargepoint Analysis dataset, from which we derive distributions for arrival, departure, and energy use, with the additional assumption of daily home charging. Given that our focus is domestic charging, which accounts for the majority of charging events, this is appropriate for the use case we study. We then use high-percentile (98th) values for daily energy and the plug-in window so that the MPC schedule remains feasible even on unusually demanding days; in this sense, the “precautionary” requirement is already embedded in the controller via conservative parameterisation.

We agree that we do not reference survey-based, stated willingness to provide manual precautionary estimates, since such surveys are not available to the best of our knowledge. However, we consider that this socio-behavioural aspect is orthogonal to our technical contribution, which is to quantify the potential carbon savings if conservative next-day energy needs are available to the controller. In practice, such precautionary estimates need not rely on user input: they can be inferred from past charging/driving data (e.g. high-percentile recent daily energy use, possibly conditioned on weekday/season) and exposed as a default that users can override when planning unusually long trips. Incentive aspects are already discussed in Section 4.2, where we point to emission-trading / pooling mechanisms as natural ways to encourage “virtuous” charging behaviours. Designing and empirically testing such schemes is beyond the scope of the present technical paper and is identified as future work.

**Author action**

We have (i) clarified in Sections 3.2. and 3.3. that plug-in behaviour and daily energy use are calibrated from the UK Electric Chargepoint Analysis dataset and implemented via conservative high-percentile assumptions, and (ii) added a short explanation in the daily-energy section that, in a practical deployment, precautionary next-day energy requirements can be learned from historical data and overridden by the user. We also slightly rephrased the end of Section 4.2 to make explicit that the design and empirical evaluation of incentive mechanisms is left for future research.

**7. The response to reviewers is addressed, but some figures still lack units or scales. Update all visuals for clarity.**

**Author response**

We appreciate the reminder on graphical clarity. We carefully reviewed all figures to ensure consistency of axes, labels, and units.
